# Beta phase synchronization in the frontal-temporal-cerebellar network during auditory-to-motor rhythm learning

**DOI:** 10.1038/srep42721

**Published:** 2017-02-22

**Authors:** Kouki Edagawa, Masahiro Kawasaki

**Affiliations:** 1 Department of Intelligent Interaction Technology, Graduate School of Systems and Information Engineering, University of Tsukuba, 1-1-1, Tennodai, Tsukuba-shi, Ibaraki 305-8573, Japan; 2 Rhythm-based Brain Information Processing Unit, RIKEN BSI-TOYOTA Collaboration Center, 2-1, Hirosawa, Wako-shi, Saitama, 351-0198, Japan

## Abstract

Rhythm is an essential element of dancing and music. To investigate the neural mechanisms underlying how rhythm is learned, we recorded electroencephalographic (EEG) data during a rhythm-reproducing task that asked participants to memorize an auditory stimulus and reproduce it via tapping. Based on the behavioral results, we divided the participants into Learning and No-learning groups. EEG analysis showed that error-related negativity (ERN) in the Learning group was larger than in the No-learning group. Time-frequency analysis of the EEG data showed that the beta power in right and left temporal area at the late learning stage was smaller than at the early learning stage in the Learning group. Additionally, the beta power in the temporal and cerebellar areas in the Learning group when learning to reproduce the rhythm were larger than in the No Learning group. Moreover, phase synchronization between frontal and temporal regions and between temporal and cerebellar regions at late stages of learning were larger than at early stages. These results indicate that the frontal-temporal-cerebellar beta neural circuits might be related to auditory-motor rhythm learning.

Aspects of dancing and music can require encoding, retaining, retrieving, imitating, and reproducing rhythms, which are defined as temporal units, tempo, beats, and the pattern of time length between the onsets of stimulus presentation and next stimulus presentation^1^. Learning of rhythm is proposed to be associated with extended brain regions such as the frontal, motor, and cerebellar areas^2^. Studies have identified the prefrontal and tactile regions as being involved in processing time, the premotor areas for processing tempo, the auditory areas for processing rhythmic patterns^1^, and the auditory-motor areas for processing beats^3^. Moreover, prefrontal-parietal-cerebellar neural circuits play important roles in rhythm encoding and memory^4^,^5^, while the cerebellum and motor areas are proposed to be involved in reproducing rhythms^6^,^7^,^8^. These studies have compared brain activity of well-trained individuals when listening to rhythms as well as that from naïve participants.

When learning a new rhythm, planning and executing the new rhythmic motions requires feedback that encodes the difference between the auditory input and the motor output of the rhythms (i.e., errors)^9^,^10^. Studies have attempted to identify brain activity underlying these rhythm-learning functions using event-related potentials (ERPs) found in electroencephalographic (EEG) signals. For instance, frontal areas have been reported to show error-related negativity (ERN), an ERP component that occurs about 50 ms after errors in rhythm production are detected^11^ as well as cognitive control^12^. However, the location of brain activity that represents the ability to learn rhythms is still unclear.

In particular, because the studies mentioned above focused on local brain activity, the role of global brain activity in perceiving and learning rhythms remains unknown. Varela, *et al*.^13^ proposed that global brain networks can be identified by analyzing the EEG phase synchronization between the distinct brain areas^13^. Numerous studies that used time-frequency analyses of human EEG data have shown that several oscillatory phases are synchronized between task-relevant brain areas^14^,^15^,^16^.

Here, we used time-frequency analyses of ERP data to clarify the roles of both local brain activity and global brain networks in rhythm learning. In particular, we focused on differences in brain activity between those who could learn a rhythm and those who could not. We analyzed EEG data that was recorded during an auditory-to-motor rhythm-reproducing task and compared the findings based on the behavioral results.

## Material and Methods

### Participants

Fourteen right-handed participants with normal or corrected-to-normal vision were tested (six female, mean age: 23.6 ± 1.3 years). All participants gave written informed consent before participation. The study was approved by the Faculty of Engineering, Information and Systems, Research Ethics Committee of the University of Tsukuba in accordance with the Declaration of Helsinki.

### Task

Participants were asked to sit in a sound shield room and refrain from moving as much as possible. Throughout the experiment, participants wore headphones with their eyes closed. The task required participants to memorize rhythms that were defined by sequences of nine sounds and the eight intervals between them. A beep was presented at the beginning of each trial to prepare participants for memorization, and was followed by the nine-sound rhythm presented through the headphones (the encoding period). A 2-s rehearsal period followed the rhythm, and a second beep was presented to signal the start of the reproduction period. Rhythms were reproduced by tapping a key on a keyboard. Each tap produced a sound, providing auditory feedback. After participants finished tapping the pattern, a third beep was presented to indicate that the trial was over. Trials using the same rhythms were repeated 13 times, which was defined as a sequence. Participants were asked to increase their accuracy as best they could as a sequence progressed.

**Figure 1:**
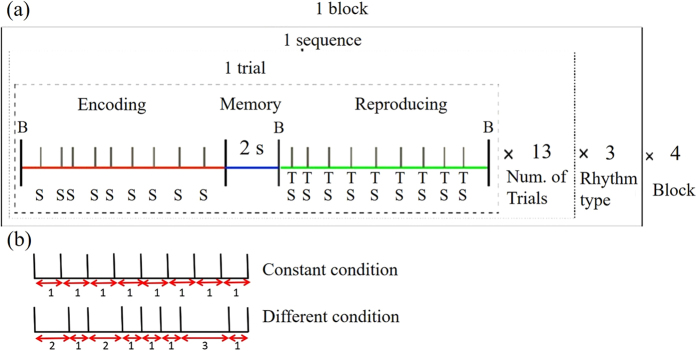
(**a**) Sample experimental procedure (13 trials × 3 rhythm types × 4 Blocks). “S”, “B”, and “T” indicate the timing of the tones, beep sounds, and finger taps, respectively. Red, blue, and green lines delineate the encoding, memory, and reproduction periods. (**b**) Example rhythms of constant (top) and variable (bottom) conditions.

All participants completed 4 blocks of 3 sequences, with each sequence using 1 of 12 different rhythms (Fig. 1). Two were constant rhythms in which the 8 intervals were equal in duration (constant rhythm condition). The other 10 rhythms had intervals that varied in duration (variable rhythm condition). Intervals in the variable rhythm conditions had one interval of either 0.3s or 0.45s, with the other 7 intervals being 2, 3, or 4 times this minimum interval. This was done because a psychological study has reported that listeners perceive and feel time intervals with integer ratios as rhythmic accents^17^. These time intervals were randomly presented in a trial.

### Behavioral analysis

Behavioral performance was evaluated by the Get-Into-Rhythm (GIR) index, which has been used in other studies^4^,^18^. The GIR index was estimated by a formula that incorporated tap onset asynchrony (TOA), which was defined by the tapping interval, and stimulus onset asynchrony (SOA), which was defined by the stimulus interval:


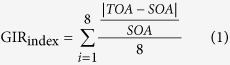


Trials in which the total number of taps did not equal nine were removed from the behavioral and EEG analyses.

### EEG recordings

EEGs were continuously recorded using 62 scalp electrodes (Ag/AgCl) embedded in an EasyCap, in accordance with the extended version of the international 10⁄20 system. EEG data were recorded using SynAmp2 (Neuroscan, El Paso, TX, USA) at a sampling rate of 1000 Hz. Reference electrodes were placed on the left and the right mastoids and were virtually connected. Eye blinks and vertical eye movements were monitored by electrodes that were placed above and below the left eye. Horizontal eye movements were measured using electrodes that were placed 1 cm horizontally from the right and left eyes. EEG data were filtered in the bandpass range of 0.1 Hz to 30 Hz.

### EEG analysis

EEG data were analyzed with MATLAB. EEG data for the reproduction period were segmented into 0.5-s epochs from 0.1-s before and 0.4-s after the onset of each tap. We analyzed the EEG epochs for each rhythm and each learning stage separately. EEG data were filtered with a 2.5 Hz high-pass filter and 50 Hz notch filter. In the ERP analyses, the peak value of the ERN component was calculated as the mean value of the EEG data between 0 and 50 ms after tone/tap onset at the frontal electrode site (Fz). Grand average topographies were created for all conditions.

To evaluate the time-frequency amplitudes and phases, we conducted wavelet analyses using Morlet’s wavelets function w(t, f) with the time domain (SD σ_t_) and the frequency domain (SD σ_f_) around their central frequency (f)^19^.


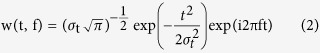


The time-frequency amplitude E(t, f) for each time point of each trial was the square norm of the result of the convolution of a complex wavelet w(t, f) with the original EEG signals s(t).


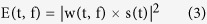



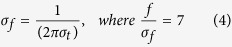


To identify the phase relationship between any two electrodes, we calculated a phase synchronization index (PSI)^20^,^21^ between electrode pair (j, k) as:


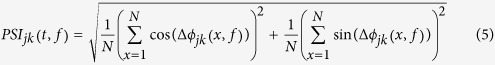


where N is the time points for the time window (i.e., N = 500 in case of 0.5-s time window) and 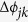 is the phase differences between two electrodes.

In the ERP, time-frequency amplitude, and PSI analyses, data were corrected by subtracting a 500-ms pre-stimulus baseline (EEG data from −2.25s to −1.75s before the onset of stimulus presentation in each trial).

In order to localize the datialed generator of the scalp EEG oscillations, we applied a standarized low resoluation EEG tomography (sLORETA)^22^. The sLORETA images were corresponded by 5 mm spatial resolution and the statistical contrast maps between conditions were calculated. The peak Talairach Atlas coordinates were identified for the ERN and beta activities in this study.

## Results

### EEG analysis

**Figure 2:**
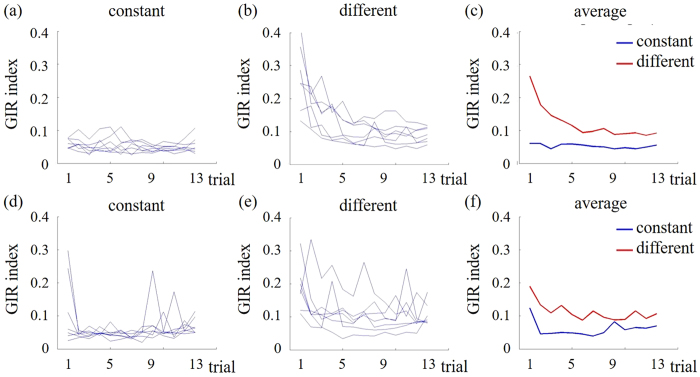
Performance of the Learning (top) and No-learning (lower) groups. Individual GIR indices for each trial are shown for the constant (**a,d**) and variable (**b,e**) conditions. Participant-averaged GIR indices for each trial are shown for the constant (blue) and variable (red) conditions in the Learning (**c**) and No-learning (**f**) groups.

Figure 2 shows the individual GIR indices for the constant (2a and d) and variable (2b and e) rhythm conditions. For each participant, a 2-way ANOVA with factors of Trial in Sequence (1 to 13) and Condition (constant or variable) was conducted. Seven participants showed a significant main effect (*Ps* < 0.05) of Trial in Sequence, indicating that they learned to produce the rhythms over time. In contrast, the other seven participants did not any main effect of Trial in Sequence, indicating that they did not learn to produce the rhythms. Based on this behavioral performance, we divided the participants into Learning and No-learning groups. The average GIR indices for the two groups in the constant and variable rhythm conditions are shown in Fig. 2c and f. GIR indices were significantly lower in the late learning stage (trials 11–13) than in the early learning stage (trials 1–3) for the Learning group, but not the No-learning group. Therefore, we compared EEG data during the reproduction periods of the early and late stages.

### ERP analysis

**Figure 3:**
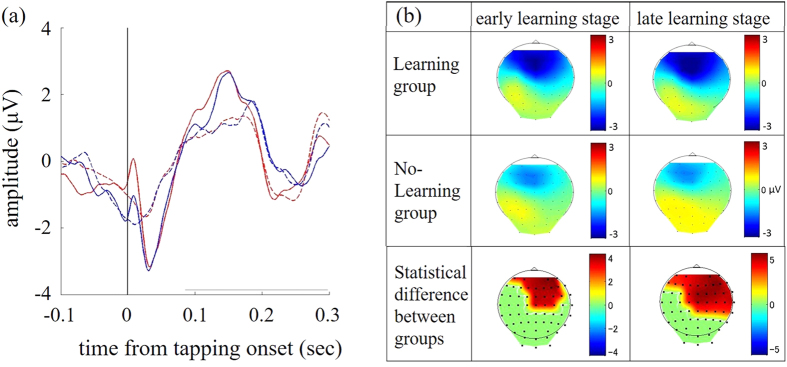
(**a**) The event-related brain potential on the frontal electrode (Fz) at the early stages (red lines) and late stages (blue lines) for the Learning (thin lines) and No-learning (dotted lines) groups. (**b**) Topographical maps showing the ERN (0–50 ms after tapping onset) at the early (left) and late (right) stages for the Learning (top) and No-learning (middle) groups. Color bars depict ERN amplitudes. Bottom topographical maps show the statistical ERN differences between groups at the early (left) and late (right) stages. Color bars depict the z-values. The absolute ERN amplitudes of Learning group was significant higher than that for the No-learning group.

In the variable rhythm condition, the ERN in the frontal area was found around 30 ms after tapping onset (Fig. 3a). Topography results showed that the mean ERN 25–35 ms after tapping onset was localized at the frontal areas in both the early and late learning stages (Fig. 3b). Although the frontal ERN was observed in both the Learning and No-learning groups, its amplitude was significantly higher in the Learning group (*P* < 0.05; Mann-Whitney U-test with Bonferroni correction; Fig. 3c). This tendency was observed without any significant difference between learning stages of both groups (early vs. late stage: *Ps* > 0.05). The ERN was not observed in the constant rhythm condition.

### Oscillatory amplitude analysis

**Figure 4:**
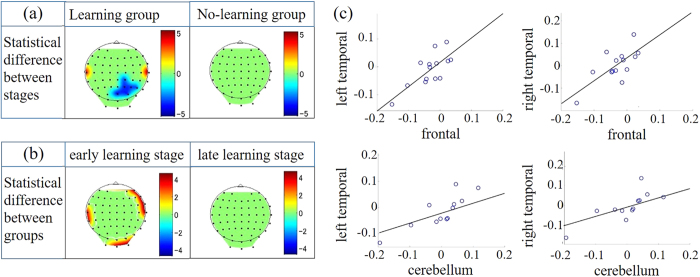
(**a**) Topographical maps showing the statistical differences in beta amplitude between stages in the Learning (left) and No-learning (right) groups. Color bars depict the z-values. The absolute amplitudes at early stages were significant higher than those at late stages. (**b**) Topographical maps showing the statistical difference in beta amplitude between groups at the early (left) and late (right) stages. Color bars depict the z-values. The absolute beta amplitudes of the Learning group were significant higher than those of the No-learning group. (**c**) Scatter plots of the individual beta amplitudes for frontal and left-temporal areas (top left), frontal and right-temporal areas (top right), cerebellum and left-temporal areas (bottom left), and cerebellum and right-temporal areas (bottom right).

Time-frequency analyses showed that the beta (21–30 Hz) amplitudes were enhanced for 0–50 ms after tapping onset in the variable rhythm condition but not the constant condition. To evaluate the effect of learning the rhythms, we compared beta amplitudes between the early and late stages of learning in each group in the variable rhythm condition. Topographical maps of the statistical z values for the Learning group showed that beta amplitudes in the temporal areas were significantly higher in the early stages of learning (*P* < 0.05; Mann-Whitney U-test with Bonferroni correction; Fig. 4a). The same comparison yielded no significant differences in the No-learning group (Fig. 4b).

Next, we compared beta amplitudes between groups at each of the two stages of learning in the variable rhythm condition. Topographical maps of the statistical z values at the early learning stages showed that beta amplitudes for the Learning group were significantly larger than those for the No-learning group in the cerebellum and right and left temporal areas (*P* < 0.05; Mann-Whitney U-test with Bonferroni correction; Fig. 4c). The same comparison yielded no significant differences in any brain area during the late stages of learning. The above beta comparisons were not found in the constant rhythm condition (all *Ps* > 0.05).

### Source analysis for ERN components and beta amplitudes

**Figure 5:**
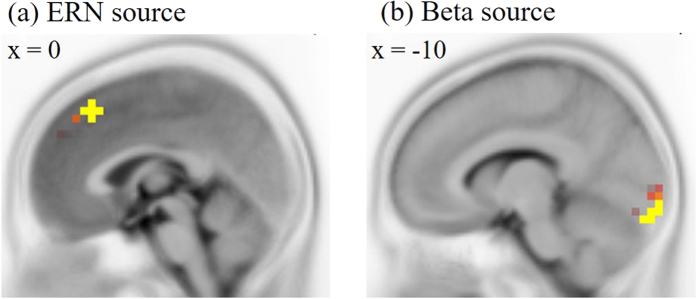
Sagittal view of brain showing source locations of the ERN amplitude (**a**) and the beta amplitudes (**b**).

In the variable rhythm condition, the source of the ERN components was identified at the medial frontal lobe (Fig. 5a; peak Talairach Atlas coordinates; x = 0, y = 30, z = 45), which is close to previous findings^23^. Moreover, the beta source was identified the cerebellum (Fig. 5b; peak Talairach Atlas coordinates; x = −10, y = −100, z = −20). When the thresholds were mild, the beta sources were also appeared at the inferior temporal lobe which is close to the auditory cortex (peak Talairach Atlas coordinates; x = ± 60, y = −40, z = −10).

### Oscillatory amplitude -correlation analysis

We conducted correlation analyses of the beta amplitudes between the brain areas for which the ERN and beta amplitudes differed between learning groups in the variable rhythm condition. We found significant positive correlations at the late stages of learning between frontal areas and the cerebellum (*r* = 0.64, *P* < 0.05), frontal and left temporal areas (*r* = 0.75, *P* < 0.05), frontal and right temporal areas (*r* = 0.71, *P* < 0.05), cerebellum and left temporal areas (*r* = 0.71, *P* < 0.05), and between cerebellum and left temporal areas (*r* = 0.73, *P* < 0.05). However, no correlation was significant at the early stages (all *Ps* > 0.05). Moreover, no correlation was significant in the constant rhythm condition (all *Ps* > 0.05).

### Correlation analysis between EEG activities and rhythm learning performance

**Figure 6:**
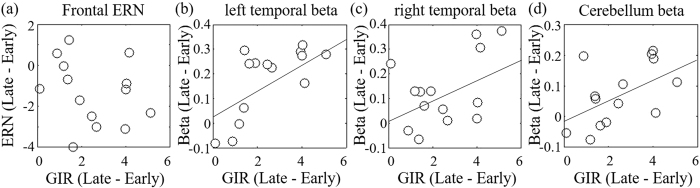
Scatter plots of the individual different GIR index between the early and the late stages and different EEG activities between the early and the late stages; (**a**) frontal ERN, (**b**) left temporal beta amplitudes, (**c**) right temporal beta amplitudes, and (**d**) cerebellum beta amplitudes.

In order to clarify the relationships between the EEG activities on the rhythm learning, we additionally conducted correlation analyses between the individual EEG and the individual performance. The learning performance was defined as the different GIR index between the early and the late stages. The results showed that the different frontal ERN amplitudes between the early and the late stage were not significantly correlated with the learning performance in the variable rhythm condition (Fig. 6a; r = −0.24, p = 0.41). Which suggested that the ERN was not directly involved in rhythm learning. In contrast, the different beta enhancements were significantly and positively correlated with the learning performance in only the variable rhythm condition (Fig. 6b–d; left temporal areas: r = −0.70, p < 0.01; right temporal areas: r = −0.46, p < 0.05; cerebellum: r = −0.54, p < 0.05).

### PSI analysis

**Figure 7:**
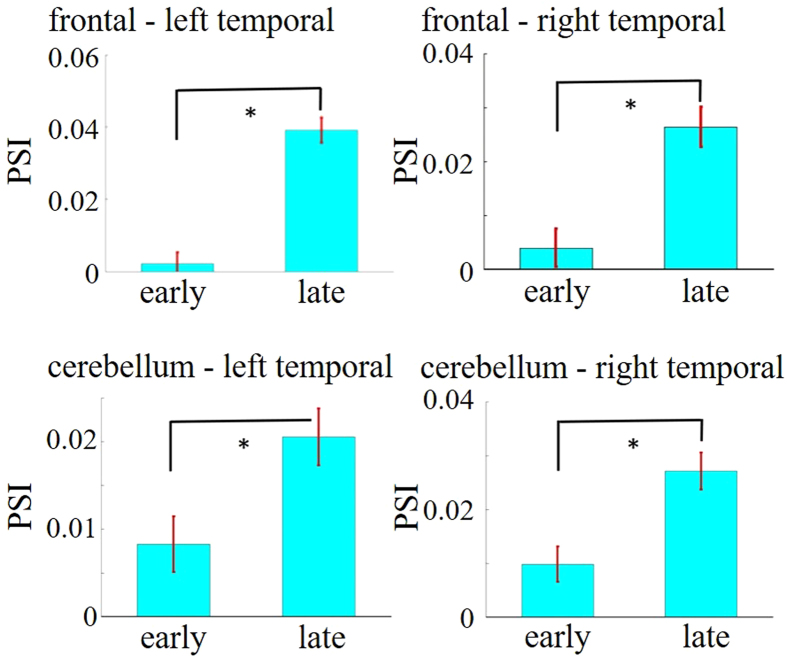
The averaged PSI values between frontal and left-temporal areas (top left), frontal and right-temporal areas (top right), cerebellum and left-temporal areas (bottom left), and cerebellum and right-temporal areas (bottom right) at early and late stages. Error bars are standard error of the mean. (^*^P < 0.05; Mann-Whitney U-test with Bonferroni correction).

Similar to the correlation analyses, PSI analyses were conducted for the beta band, comparing brain areas in which the ERN and beta analyses yielded differences between learning groups in the variable rhythm condition. We compared the normalized PSI between the early and late stages of learning and found that in the Learning group, several pairs of brain regions showed significantly higher synchrony during the late stages (Fig. 7). These included frontal areas and the cerebellum (*z* = 5.02, *P* < 0.05), frontal and left temporal areas (*z* = 7.43, *P* < 0.05), frontal and right temporal areas (*z* = 3.83, *P* < 0.05), cerebellum and left temporal areas (*z* = 2.32, *P* < 0.05), and cerebellum and left temporal areas (*z* = 3.48, *P* < 0.05). In contrast, no comparison of learning stages in the No-learning group yielded any significant differences in beta PSI between brain areas (all *Ps* > 0.05). Any comparison was not significant in the constant rhythm condition (all *Ps* > 0.05).

## Discussion

This study suggests that brain networks among the frontal, temporal, and cerebellar cortices are involved in rhythm learning. We used a repeated auditory-motor rhythm-reproducing task and compared EEG data from early and late learning stages in and between individuals who were able to learn the rhythms and those who could not. EEG analyses showed that frontal error prediction-related activity and beta oscillatory activity among extended brain regions play an important role in facilitating learning to produce rhythms.

Frontal ERN amplitudes in the Learning group, but not the No-learning group, were enhanced when reproducing the rhythms. This ERP component was only observed in the frontal areas, which is consistent with other studies showing that the ERN is generated from the midline frontal cortex, specifically, the anterior cingulate cortex and pre-supplementary motor area^24^,^25^. The frontal ERN is thought to be associated with top-down control mechanisms^12^; error monitoring^26^, error adaptation^27^,^28^, and construction of internal models of audio-motor association^29^. These two functions were proposed to play an important role in several types of learning. However, the frontal ERN in this study did not differ significantly between the stages, indicating that it did not change as the task was learned. Rather, its amplitude was higher in the Learning group. Thus, our results along with the previous findings suggest that properties of the frontal ERN might depend on the ability of individuals to learn the rhythms.

Past studies have reported the ERN components which appear after errors. In contrast, the ERN in this study was observed after every tapping even as it was either synchronized or desynchronized with the correct rhythm. However, this study is not able to separate the correct and error tapping, because the correct and error was evaluated by whole tapping rhythmic sequences (i.e. 9 tapping) but not by one tapping. Therefore, the ERP component in this study might not be exactly same as the ERN in previous studies. This results suggested that the ERP component would be related to the detection of errors in the whole rhythmic sequences as well as one error.

Beta oscillatory activity increased in the right and left temporal areas during the rhythm-learning task. For those who learned to produce the rhythms, greater beta activity was observed at the early stages of learning in the variable rhythm condition—the time when unfamiliar rhythms are acquired. In contrast, enhancements were not observed when this type of learning was not occurring (i.e., the late learning stage for the Learning group and both stages for the No-learning group). Beta oscillations have been shown to be associated with movement, including initiation of movements^30^, movement execution^31^, voluntary movements^32^, and acquisition of new movements^33^. Further, in addition to auditory processing, other studies have reported that decreased beta amplitudes in temporal areas are related to rhythmic movements^34^,^35^,^36^,^37^. Moreover, the temporal areas are thought to play important roles in rhythm reproduction, thorough interactions with the frontal areas and the basal ganglia, thus forming connections between the auditory stimulus and the motor response^38^.

The beta amplitudes in the cerebellum were also higher at the early learning stage in the Learning group. However, difference in cerebellum amplitudes were not observed when comparing the early and late stages within the Learning group. Evidence from monkey physiological studies, human fMRI studies, and lesion studies indicate that the cerebellum is associated with the rhythm-information processing^2^,^39^,^40^,^41^,^42^. Moreover, human EEG studies have shown beta oscillations in the cerebellum during sensorimotor information processing^43^,^44^. Thus, our results support these findings and suggest that beta oscillatory activity in temporal auditory areas and the cerebellum are involved in the acquisition of unfamiliar rhythms during rhythm learning.

Here, the commonly shared beta-amplitude modulation is evidence that beta oscillations play an important role in auditory-to-motor rhythm learning by functionally connecting the temporal and cerebellar areas. The significant beta-amplitude correlation between the two areas is what led to their significant beta-phase synchronization during rhythm-acquisition periods (the early learning stage in the learning group). Indeed, as mentioned above, studies have shown that these brain areas are involved in sensorimotor processing. Furthermore, a monkey physiological study has shown that the cerebellum affects the prefrontal cortex in the planning and learning of movements^45^. The frontal-cerebellar networks, along with the parietal and motor areas, are proposed to be related to the motor control^4^,^46^. In addition to the motor control, our rhythm-learning task needs error adjustments and the integration of auditory information processing and motor control. Therefore, through the beta oscillations, the temporal auditory areas were correlated and synchronized with both the cerebellum and the frontal areas, which showed the ERN. Taken together, synchronization of the frontal-cerebellar beta networks is a possible neural mechanism for auditory-to-motor rhythm learning.

Because the channels representing beta enhancement in the temporal and occipital areas are all at the edge of recording space, they might be possibly due to a common artifact. However, sLORETA analysis localized the beta sources at the auditory areas and cerebellum. Moreover, these beta enhancements and interactions were not observed in the late stage of the variable rhythm condition and in the constant rhythm condition which was positioned as the control condition. Therefore, the beta activities and interactions in this study were significant.

This study has a limitation in that detailed locations within the brain could not be identified owing to the low spatial resolution of EEG. In particular, dissociating activity in the cerebellum from that in the visual cortex was difficult because these locations are quite close. We placed the three cerebellar electrodes 1 cm below the visual electrodes (O1, Oz, and O2 electrodes) to measure cerebellar activity. In the occipital areas of this study, the cerebellar electrodes, but not the visual electrodes, showed the significant differences between groups at the early stage. The occipital modulations were not related to visual processing, as the task required no visual input and participants sat with their eyes closed. Thus, although we confirmed that activity differed between the cerebellum and visual cortices, future studies with combined fMRI-EEG measurements should be conducted to clarify whether or not the region we observed here was truly the cerebellum.

## Additional Information

**How to cite this article**: Edagawa, K. and Kawasaki, M. Beta phase synchronization in the frontal-temporal-cerebellar network during auditory-to-motor rhythm learning. *Sci. Rep.* **7**, 42721; doi: 10.1038/srep42721 (2017).

**Publisher's note:** Springer Nature remains neutral with regard to jurisdictional claims in published maps and institutional affiliations.
